# Prevalence and molecular characterization of *Staphylococcus aureus* from human stool samples

**DOI:** 10.1186/s13756-018-0331-3

**Published:** 2018-03-20

**Authors:** A. E. Kates, D. Thapaliya, T. C. Smith, M. L. Chorazy

**Affiliations:** 1 Division of Infectious Disease, Department of Medicine, School of Medicine and Public Health, University of Wisconsin-Madison, Madison, WI USA; 2 Department of Biostatistics, Epidemiology and Environmental Health Services, College of Public Health, Kent State University, Kent, OH USA; 3 Department of Epidemiology, College of Public Health, University of Iowa, Iowa City, IA USA

**Keywords:** Methicillin-resistant *Staphylococcus aureus*, Hospitals, Antibiotic resistance, Intestinal carriage

## Abstract

**Background:**

To determine the prevalence of intestinal *S. aureus* colonization of patients at a large teaching hospital and determine the molecular characteristics of the identified strains. The second objective of this research was to determine risk factors associated with *S. aureus* intestinal colonization.

**Methods:**

A cross-sectional study of 781 specimens from inpatients and outpatients at the University of Iowa Hospitals and Clinics Clinical Microbiology Laboratory was conducted. *S. aureus* was identified using traditional culture methodologies. Methicillin-resistance was determined via PCR of the *mecA* gene. PVL PCR, *spa* typing, and antimicrobial sensitivity testing were also done. A nested case-control study was done on a subset of patients with all colonized patients defined as cases and non-colonized controls. Medical record abstractions were done to identify risk factors for intestinal colonization in the nested study.

**Results:**

Out of 625 patients included in the final study, 58 were positive for *S. aureus* (9.3%). One isolate was positive for the PVL gene. A high number of isolates were resistant to multiple antibiotics including oxacillin (43.1%), erythromycin (51.7%), and levofloxacin (41.4%). All isolates were susceptible to vancomycin, daptomycin, linezolid, and quinupristin-dalfopristin. In the nested study, having a disease or condition of the gastrointestinal tract significantly increased the odds of intestinal colonization (OR: 1.96, 95% CI: 1.04–3.7; aOR: 13.9, 95% CI: 1.67–115.7). No other variables were significantly associated with increased odds of colonization.

**Conclusions:**

*S. aureus* was identified from the stool of patients at the University of Iowa Hospitals and Clinics, with a large number of those isolates being resistant to antibiotics and may serve a reservoir for subsequent infections as well as asymptomatic transmission.

## Background

*Staphylococcus aureus* is a commensal bacterium and important cause of healthcare-associated infections [1]. Nasal carriage is considered to be the most important site of *S. aureus* colonization [2] and is the best-studied [3]. However, other extra-nasal body sites, including the gastrointestinal tract, are known to harbor *S. aureus* [4–6]. Recent studies have found *S. aureus* in the intestines of healthy humans [7, 8] as well as the intestines of hospitalized patients [9].

*S. aureus,* an in particular methicillin-resistant *S. aureus* (MRSA) intestinal colonization, may be more common than previously thought and has been shown to be clinically important [10, 11]. Rectal carriers have been found to be at increased risk of developing *S. aureus* infections [10] and gastrointestinal carriage of MRSA has been associated with nosocomial antibiotic-associated diarrhea [11]. Screening for gastrointestinal carriage has been shown to identify colonized patients who would have been otherwise missed [12]. *S. aureus* gastrointestinal carriage may be an overlooked reservoir, contributing to hospital infection and transmission.

The objectives of this study were to determine the prevalence of *S. aureus* and MRSA in human stool samples at a large university hospital, to characterize the identified isolates by molecular methods, and to assess potential risk factors for intestinal carriage. We hypothesized *S. aureus* and MRSA prevalence in stool will be similar to what has been reported previously in the literature [3]. Furthermore, we hypothesized having a gastrointestinal condition would increase the risk of intestinal *S. aureus* carriage.

## Methods

### Population and design

We conducted a cross-sectional study of 781 stool specimens retrieved from the University of Iowa Hospitals and Clinics (UIHC) Clinical Microbiology Laboratory between September 2010 and March 2011. UIHC is Iowa’s only comprehensive academic medical center, consisting of 811 beds. Though patients are primarily drawn from eastern Iowa, the hospital serves the entire state and into the region beyond (including parts of Illinois, Wisconsin, Minnesota). The specimens were provided from a bank of existing biologic specimens originally collected for the purpose of patient clinical care as part of standard clinical practice. Samples remaining after completion of diagnostics that would have otherwise been disposed by the Clinical Microbiology Laboratory were collected by the researchers and stored at − 80 **°**C for further analysis. This convenience sample included both inpatients and outpatients from Iowa, Northern Missouri, and Western Illinois. A nested case-control study was conducted using electronic patient medical records to assess potential risk factors for *S. aureus* intestinal carriage. All patients positive for intestinal *S. aureus* carriage were included in the nested study and considered cases. Two controls were chosen for every case based on admission date and time of stool sample collection within ± 24 h. Only one stool sample per patient was included. If more than one stool sample per patient was included in the biobank, only the first stool provided by the patient was included in this analysis.

### Ethics, consent, and approval

The University of Iowa Institutional Review Board approved all study protocols.

### Identification and characterization of *S. aureus*

50 μL of stool was plated onto Baird-Parker Agar and CHROMagar MRSA media (Becton Dickinson and Company, Sparks, Maryland, USA) and incubated for 48 h at 35 **°**C. Presumptive positive colonies were streaked onto Columbia CNA with 5% sheep blood (Becton Dickinson and Company, Sparks, Maryland, USA), and incubated for 24 h at 35 **°**C. All isolates were tested for *S. aureus* using the catalase, coagulase, and Pastorex Staph Plus rapid latex agglutination (Bio-Rad, Redmond, Washington, USA) tests. Any patient who was culture positive for *S. aureus* and/or MRSA in the stool was considered intestinally colonized.

All *S. aureus* isolates were tested for antimicrobial susceptibility using broth dilution as described by the Clinical and Laboratory Standards Institute [13]. Isolates were tested for susceptibility to oxacillin, tetracycline, erythromycin, clindamycin, trimethoprim-sulfamethoxazole, gentamycin, levofloxacin, vancomycin, daptomycin, quinupristin/dalfopristin, linezolid, and rifampin.

Whole, genomic DNA was extracted using the Wizard Genomic DNA purification kit (Promega Corporation, Madison, Wisconsin, USA) adapted for *S. aureus*. Presence of the *mecA* [14] and PVL [15] genes were determined through end-point PCR. *spa* typing was carried out using the primers and methodologies described by Ridom Bioinformatics and sequences were interpreted utilizing the Ridom StaphType software (Ridom GmbH, Würzburg, Germany). For phenotypically MRSA isolates negative for the *mecA* gene, the presence of the *mecC* gene was determined [16]. All molecular procedures were carried out using known positive and negative controls, including USA300 (*mecA*, PVL) and LGA251 (*mecC*).

### Medical record abstraction

Medical record abstractions were done on all *S. aureus* and MRSA-positive patients (cases) and two culture-negative patients (controls). Controls were chosen based on date of admission and time of sample collection relative to cases. Demographic information (gender, age, and race/ethnicity), reason for specimen collection, hospital admission and discharge dates, ICU admission and discharge dates, death date, co-infections, recent antimicrobial use, recent anti-motility medication use, history of gastrointestinal disorders, and history of immunosuppressive conditions were abstracted from the electronic medical record.

### Statistical analysis

Statistical analyses were performed using SAS, version 9.3 (SAS Institute, Cary, NC). There were 781 banked samples available to the investigators from the UIHC. Two controls for each case was decide upon because it has been shown more than two controls per case does not greatly increase the statistical power of the analysis [17]. Logistic regression was performed to assess risk factors for intestinal colonization. The primary exposure of interest, having a disorder of the gastrointestinal tract, was modeled as dichotomous (yes/no). Backwards selection was used to determine which variables to include into the model (*p* > 0.2 threshold to stay in the model). Interaction terms were assessed between all covariates. If the term was significantly associated with *S. aureus* infections, it was included into the final model and the Akaike information criterion (AIC) was assessed. If the model including the interaction term increased the AIC by more than three over the model without the term, the interaction term was not included in the final model. Odds ratios and 95% Confidence Intervals were calculated and a significance level of *p* = .05 was used.

## Results

**Fig. 1:**
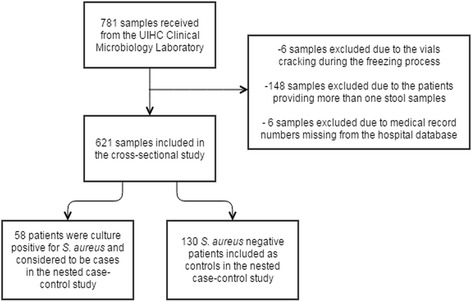
Flow chart of the participant inclusion and exclusion criteria

Of the 781 samples retrieved from the UIHC Clinical Microbiology Laboratory, six samples were excluded due to the tubes breaking during the freezing process. An additional 148 samples were excluded due to patients providing more than one stool sample and six additional samples were excluded due to medical records that were not able to be located, leaving 621 patients included in the study (Fig. 1). Demographic and clinical characteristics of the 621 patients included in the study are described in Table 1. The average age for all patients was 51.6 years (stan. Dev.: 19 years, range: 0 to 94 years). A majority of patients were female (320, 51.5%) and Caucasian (560, 90.2%). A majority were inpatients (487, 78.4%) with 20.1% (126/625) being outpatients, and 1.3% (8/625) being housed outpatients. The average length of stay for inpatients as defined as the entire time they were admitted for the stay when their stool samples was collected was 15.8 days (stan. Dev.: 28.6 days). There were no significant differences between carriers and non-carriers for any variables listed in Table 1.

**Table 1:** Patient demographic data by *S. aureus* intestinal colonization status

	Carriers *n* = 58	Non-carriers *n* = 567	*P*	Total *n* = 621
Age (Mean years ± Std. dev.)	51.2 (±20.4)	51.6 (±18.9)	0.897	51.6 (±19.0)
Sex				
Male	24 (41.4%)	277 (49.2%)		301 (48.5%)
Female	34 (58.6%)	286 (50.8%)	0.256	320 (51.5%)
Race/ Ethnicity				
Caucasian	52 (89.7%)	508 (90.2%)		560 (90.2%)
African American	2 (3.5%)	24 (4.3%)		26 (4.2%)
Hispanic or Latino	3 (5.2%)	8 (1.4%)		11 (1.8%)
Other^a^	1 (1.7%)	24 (4.2%)	0.486	25 (4.0%)
Visit Type				
Inpatient	44 (75.9%)	443 (78.7%)		487 (78.4%)
Outpatient	13 (22.4%)	113 (20.1%)		126 (20.3%)
Housed outpatient^b^	1 (1.7%)	7 (1.2%)	0.865	8 (1.3%)
Length of Stay (Mean days ± Std. dev.)	21.7 (±51.7)	15.2 (± 25.1)	0.992	15.8 (± 28.6)

^a^Other includes American Indian or Alaskan Natives, Asian, Native Hawaiian or Pacific Islander, as well as individuals who declined to provide a race or ethnicity

^b^Housed outpatient refers to patients being held for observation

Of the 621 samples, 58 (58/621, 9.3%) were positive for *S. aureus,* 26 of which (26/621, 4.2%) were MRSA via the presence of the *mecA* gene, with an MRSA prevalence of 44.8% (26/58). One isolate was positive for PVL. Thirty unique *spa* types were identified, with t002 being the most prevalent at 66.7% (*n* = 20) followed by t012 and t1635 both at 10% (*n* = 3). The BURP analysis resulted in one major cluster with the hospital-associated strain t002 as the founder. The *spa* cc-002 grouping accounted for 24 strains (42% of all strains) and four *spa* types (13% of all *spa* types). Two *spa* types (three isolates) were excluded as they had less than 5 repeat sequences present in the *spa* gene.

**Fig. 2:**
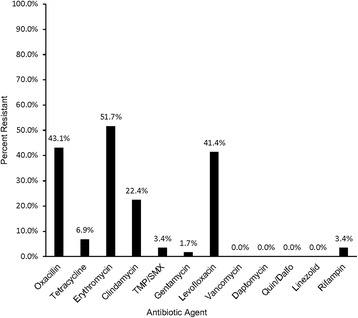
Antibiotic susceptibility testing using minimum inhibitory concentrations (*n* = 58)

**Fig. 3:**
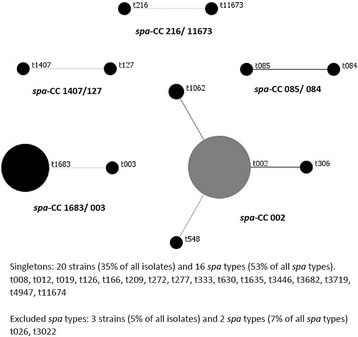
Based Upon Repeat Pattern Analysis of all positive *S. aureus* isolates (*n* = 58). Based Upon Repeat Pattern (BURP) analysis was used to group all 58 *S. aureus* isolates into cluster complexes. Cost distances of ≤4 were used to define clusters and *spa* types with 5 or fewer repeats were excluded from the analysis

Antimicrobial susceptibility testing was performed on all isolates positive for *S. aureus* (Fig. 2). Resistance was observed for most antibiotics tested. The highest prevalence of resistance was to erythromycin at 51.7% (*n* = 37) followed by oxacillin at 43.1% (*n* = 25) and levofloxacin at 41.4% (*n* = 24). Resistance to clindamycin was observed at 22.4% (*n* = 16). No isolates were resistant to vancomycin, daptomycin, or quinupristin/dalfopristin. Resistance to all other antimicrobials was low (Fig. 3). Twenty-six (44.8%) isolates met the definition for multi-drug resistance (MDR) with having acquired non-susceptibility to at least one agent in three or more antimicrobial categories [18]. Of the MDR isolates, one isolate was resistant to at least one agent in six antimicrobial categories and one isolate was resistant to ≥1 agent in five categories. Twelve isolates were resistant to ≥1 agent in four categories and 9 isolates were resistant to ≥1 agent in 3 categories. Three isolates met the definition of MDR by being MRSA. Twenty-two of the 58 isolates (37.9%) were susceptible to all antibiotics tested. Six isolates (10.3%) were resistant to only one antimicrobial and two were resistant to two antimicrobials (3.4%). Two isolates were phenotypically resistant to oxacillin according to the AST (both isolates had a minimum inhibitory concentration [MIC] of ≥4 μg/mL); however, both isolates were negative for the presence of the *mecA* and *mecC* genes.

Medical record abstractions were completed on 189 patients (58 cases and 131 controls). A majority of cases were Caucasian (*n* = 168, 88.9%) and slightly over half were male (*n* = 98, 51.8%). In the univariate analysis, most covariates were not significantly associated with *S. aureus* intestinal colonization. We observed a borderline significant association with sex with females having increased odds of intestinal colonization, though the confidence interval included 1.0 (OR: 1.84, 95% CI: 0.98–3.44). Having a history of a gastrointestinal condition, the primary predictor, was significant in the unadjusted, univariate model with those having any disorder of the gastrointestinal tract having increased odds of intestinal colonization (OR: 1.96, 95% CI: 1.04–3.7). The most prevalent disorder of the GI tract was gastroesophageal reflux disease (GERD) at 31 of the 189 patients (16.4%) of the total population analyzed for the nested study. The second most prevalent condition was lower GI bleed (*n* = 16, 7.4%) followed by Crohn’s disease (*n* = 13, 6.9%) and any inflammatory disorder of the intestines including inflammatory bowel disorder (*n* = 13, 6.9%). No other covariates were significantly associated with intestinal colonization in the univariate analyses (Table 2).

**Table 2:** Associations between potential risk factors and *S. aureus* intestinal colonization

Risk Factor	Cases (N = 58)	Controls (*N* = 131)	Unadjusted OR (95% CI)	Adjusted OR (95% CI)
Age				
0–29 years	9	13	Ref	Ref
30–49 years	14	34	0.58 (0.020–1.65)	2.94 (0.43–19.88)
50–69 years	28	60	0.67 (0.026–1.77)	5.02 (0.81–30.9)
Over 70 years	7	23	0.44 (0.13–1.5)	1.42 (0.15–13.6)
Sex				
Male	24	74	Ref	Ref
Female	33	58	1.84 (0.98–3.44)	1.93 (0.94–3.96)
Race/ Ethnicity				
Caucasian	53	115	Ref	
African American	2	5	0.9 (0.17–4.8)	
Hispanic/ Latino	3	2	3.35 (0.54–20.63)	
Other	1	8	0.28 (0.03–2.29)	
*S. aureus* Infection^a^				
No	53	121	Ref	Ref
Yes	5	10	1.14 (0.37–3.50)	0.83 (0.19–3.54)
Other Coinfections				
No Coinfections	26	46	Ref	
One	21	60	0.62 (0.34–1.23)	
Two	4	18	0.49 (0.16–1.48)	
Three or more	7	7	0.67 (0.19–2.34)	
Collection Reason				
Abdominal pain	5	13	Ref	Ref
*Clostridium difficile*	23	37	1.61 (0.51–5.13)	1.87 (0.49–7.11)
Diarrhea	11	72	0.67 (0.21–2.14)	0.59 (0.15–2.25)
Other	19	9	2.25 (0.63–8.03)	2.74 (0.64–11.7)
Deceased^b^				
No	44	83	Ref	Ref
Yes-within 30 days	3	11	0.71 (0.18–2.8)	0.74 (0.13–4.67)
Yes- after 30 days	14	26	0.4 (0.16–0.98)	0.29 (0.1–0.86)
Undeterminable	4	7	1.08 (0.3–3.9)	0.71 (0.17–2.96)
Gastrointestinal Disease				
No	21	69	Ref	Ref
Yes	37	62	1.96 (1.04–3.7)	13.9 (1.67–115.7)
Immunocompromised				
No	15	34	Ref	
Yes	43	97	1.01 (0.47–2.04)	
ICU Stay				
No	47	98	Ref	
Yes	11	33	0.69 (0.32–1.5)	
ICU Unit				
No ICU stay	47	98	Ref	
MICU	5	21	0.5 (0.18–1.4)	
SICU	2	6	0.7 (0.14–3.58)	
Other	4	6	1.4 (0.4–5.2)	
ICU Length of Stay				
0 days	47	99	Ref	Ref
1 to 4 days	1	14	0.15 (0.02–1.18)	0.15 (0.02–1.36)
Greater than 4 days	10	18	1.18 (0.5–2.73)	1.38 (0.46–4.15)
Total Length of Stay				
0 days	12	23	Ref	
1 to 10 days	23	59	0.75 (0.32–1.75)	
11 to 29 days	10	26	0.74 (0.27–2.02)	
Greater than 30 days	13	23	1.08 (0.41–2.87)	
Antibiotics used				
No	26	40	Ref	
Yes	32	91	0.54 (0.29–1.02)	
Laxatives used				
No	43	100	Ref	Ref
Yes	15	31	1.13 (0.56–2.3)	1.94 (0.8–4.6)
Anti-motility agent used				
No	57	122	Ref	Ref
Yes	1	9	0.238 (0.03–1.92)	0.16 (0.02–1.53)

^a^*S. aureus* infections are defined as clinical infections extracted from the patients medical record

^b^Mortality is not considered a risk factor colonization. For this variable, *S. aureus* colonization is a risk factor for mortality

The final adjusted model included having a disorder of the gastrointestinal tract, age, sex, laxative usage, anti-motility agent usage, having a *S. aureus* infection, whether the patient died, ICU length of stay, and the reason for specimen collection. An interaction between having a gastrointestinal disorder and age was forced into the model as the interaction term was verging on significance (*p* = 0.058) and did not increase the AIC by more than 3. In the final model, having a disorder of the GI tract was significantly associated with increased odds of being intestinally colonized with *S. aureus*, though the confidence interval is very wide (OR: 13.9, 95% CI: 1.67–115.7). No other covariates included in the final model were significantly associated with intestinal *S. aureus* colonization (Table 2).

## Discussion

We identified *S. aureus* in the intestines of patients at UIHC and report an intestinal colonization rate of 9.3%. During the 1950’s, *S. aureus* intestinal colonization was studied in greater detail than seen in the current literature [19]. Studies from this time report carriage prevalence ranges between 8% and 30% [3, 20, 21]. While researchers have known of the relationship between intestinal *S. aureus* carriage and antibiotic-associated diarrhea, as well as the increased risk of other *S. aureus* infections, intestinal carriage has been studied far less than carriage at other anatomical sites, particularly the nares. Recent studies of *S. aureus* intestinal carriage have detected intestinal carriage rates ranging from 10% [22] to as high as 37% [10]. It has been estimated the average rate of intestinal colonization in hospitalized patients is roughly 20% [3], though the number of studies included in reaching this estimate is small and the populations vary greatly. Our observed prevalence of 9.3% is lower than the estimated average and lower than most studies have reported to date. This may be due to the fact the population screened for this study included patients from many departments across a large teaching hospital as well as outpatients seen in the emergency department. Use of frozen instead of fresh stools may also have reduced our recovery rate.

Many of the studies in recent years have focused on MRSA carriage and have reported MRSA intestinal carriage rates frequently ranging from 5% [22, 23] to 10% [24] with some studies finding a MRSA prevalence as high as 22% in high-risk populations [25]. The observed prevalence of MRSA reported here is 4.2%, which is lower than others have reported. Of the *S. aureus* isolates identified, 44.8% met the definition for MDR-SA. Two of these isolates were phenotypically resistant to oxacillin; however, they were not positive for either resistance gene – *mecA* or *mecC* –tested for.

We found one isolate to be positive for the PVL gene [26]. As PVL genes are mainly associated with community- associated *S. aureus,* it is not surprising we found such a low prevalence in clinical cohort. *Spa* type t002 was the most frequently identified *spa* type accounting for 66.7% of all identified *spa* types. This *spa* type is most frequently associated with hospital strains and typically belongs to the pulsed-field type USA100 [27]. *Spa* type t012, the second most prevalent *spa* type observed in this study (10% of all *spa* types) is frequently found in community settings and may be associated with younger age [28].

Prevalence of intestinal carriage is dependent on the method of specimen collection employed. Varying rates of colonization are seen by method and anatomical site used to define “gastrointestinal.” The two main methods used to collect specimens are swabs and stool samples. Swabs collected from the rectum, anus, perineum, and the groin or inguinal region are typically accepted as representing intestinal carriage of *S. aureus* in addition to stool specimens [3]. Several studies have assessed which method and region provide the most consistent rate of colonization, but these studies are few and do not provide a consensus. Rectal swabbing has been shown to yield more *S. aureus* than stool cultures [29]. This may explain why our study reports a lower prevalence compared to many of the studies cited above which used rectal or perineal swabbing.

Intestinal colonization may be an important reservoir for the dissemination of *S. aureus* in the health care setting. Intestinal colonization is known to lead to increased risk of infection [30–32], though the mechanism is not clear. It has been hypothesized intestinal colonization increases colonization or contamination of the skin which in turn increases contamination of the patient’s environment. Environmental contamination then increases both the risk of infection as well as the potential for nosocomial transmission [31]. While we were unable to investigate colonization of other body sites in this study, *S. aureus* intestinal colonization has been associated with an increased risk of skin colonization [10]. Nasal and intestinal carriage are also frequently observed in the same patient; however, intestinal colonization alone does increase the detection sensitivity [3, 22, 23, 33–35]. In a meta-analysis by McKinnell et al., it was found rectal screening increased yield by 20%, with rectal screening having the greatest impact in hospitals with a low MRSA prevalence (23% increase) [35].

To assess potential risk factors for intestinal colonization, we performed a nested case-control study. We hypothesized having a disease or condition of the gastrointestinal tract would put patients at increased risk of *S. aureus* intestinal colonization. In the univariate analysis we found gastrointestinal conditions did significantly increase the odds of being colonized with *S. aureus* (OR: 1.96, 95% CI: 1.04–3.7) and this association remained significant after adjusting for the other variables included in the final model (OR: 13.9, 95% CI: 1.67–115.7), though the 95% confidence interval was very wide due to a small sample size. No other variables abstracted from the medical record were significantly associated with an increased risk of intestinal colonization in either the univariate or adjusted models. Other studies have reported several potential risk factors for intestinal *S. aureus* colonization. It has been shown that a stay in an extended care facility or nursing home can significantly increase the risk of intestinal colonization [36]; however, we were unable to assess nursing home stays in the present study. Studies have also reported length of stay [37], a recent history of antimicrobial usage [37–39], a history of MRSA infection [38], and dependence on healthcare workers to perform activities of daily living [37] all significantly increasing the risk of intestinal *S. aureus* carriage. While we were not able to assess activities of daily living in the medical record, we did not detect a significant association with LOS, antibiotic usage, or the history of MRSA infections and colonization in our population. This may be due to the inclusion of a single hospital in this study, smaller sample size, and the reliance on medical record data.

Our study has several limitations. The first is the specimens analyzed were not collected for research purposes, but for routine medical care and as such we were only able to address intestinal colonization in a cross-sectional study and were unable to determine the duration of colonization in these patients. Future studies are needed to determine colonization duration, as well as the rates of persistent versus intermittent carriage. Furthermore, we were unable to determine if the *S. aureus* strain the patient was colonized with was the same strain causing an *S. aureus* infection, or otherwise present in other locations of the patient’s body or environment. This study was conducted in a single hospital in Iowa with a predominantly white population which may limit the generalizability of the study findings. However, UIHC is a large teaching hospital with a catchment area including several surrounding mid-western states*. S aureus* was identified from the stool of patients at the UIHC. Lastly, the samples used for this study were collected as part of routine clinical care and as such, all participants had some degree of gastrointestinal symptoms. A history of gastrointestinal conditions increased the risk of intestinal *S. aureus* carriage. Many of those isolates are resistant to antibiotics and may serve as a reservoir for subsequent infections and transmission events.

## Conclusions

*S. aureus* colonization was found in 9.3% of tested stool samples. Having a disease or condition of the gastrointestinal tract significantly increased the odds of intestinal colonization. A diverse array of molecular types were isolated, and antibiotic resistance was common, including methicillin resistance and multi-drug resistant strains.

## Abbreviations

AIC: Akaike information criterion
BURP: Based Upon Repeat Pattern.
GERD: Gastroesophageal reflux disease
LOS: Length of stay
MDR: Multi-drug resistant
MIC: Minimum inhibitory concentration
MRSA: Methicillin-resistant *Staphylococcus aureus*
UIHC: University of Iowa Hospitals and Clinics
